# Sympathetic nerve innervation is required for beigeing in white fat

**DOI:** 10.14814/phy2.14031

**Published:** 2019-03-14

**Authors:** Qiang Cao, Jia Jing, Xin Cui, Hang Shi, Bingzhong Xue

**Affiliations:** ^1^ School of Biology Georgia State University Atlanta Georgia

**Keywords:** Beige adipocytes, sympathetic nerve system, thermogenesis

## Abstract

It is increasingly recognized that activation of beige adipocyte thermogenesis by pharmacological or genetic approaches increases energy expenditure and alleviates obesity. Sympathetic nervous system (SNS) directly innervating brown adipose tissue (BAT) and white adipose tissue (WAT) plays a key role in promoting nonshivering thermogenesis. However, direct evidence that supports the importance of SNS innervation for beige adipocyte formation is still lacking, and the significance of beige adipocyte thermogenesis in protection of body temperature during cold challenge is not clear. Here we tested the necessity of SNS innervation into WAT for beige adipocyte formation in mice with defective brown fat thermogenesis via interscapular BAT (iBAT) SNS denervation. SNS denervation was achieved by microinjection of 6‐hydroxydopamine (6‐OHDA), a selective neurotoxin to SNS nerves, into iBAT, inguinal WAT (iWAT), or both. The partial chemical denervation of iBAT SNS down‐regulated UCP‐1 protein expression in iBAT demonstrated by immunoblotting and immunohistochemical measurements. This was associated with an up‐regulation of UCP1 protein expression and enhanced formation of beige cells in iWAT of mice with iBAT SNS denervation. In contrast, the chemical denervation of iWAT SNS completely abolished the upregulated UCP‐1 protein and beige cell formation in iWAT of mice with iBAT SNS denervation. Our data demonstrate that SNS innervation in WAT is required for beige cell formation during cold–induced thermogenesis. We conclude that there exists a coordinated thermoregulation for BAT and WAT thermogenesis via a functional cross talk between BAT and WAT SNS.

## Introduction

Obesity has become an epidemic disorder that is closely associated with a panel of metabolic diseases including insulin resistance/type 2 diabetes, dyslipidemia, cardiovascular diseases and hypertension (Hill et al. 2012). Obesity results from a prolonged energy imbalance due to excess energy intake over energy expenditure (Hill et al. 2012). While white adipose tissue (WAT) serves as the major depot for energy storage, brown adipose tissue (BAT) instead functions to dissipate energy as heat through nonshivering thermogenesis, in which oxidative phosphorylation is uncoupled from ATP synthesis by the inner mitochondrial membrane protein called UCP1 (Cannon and Nedergaard 2011). Despite the distinct metabolic function between WAT and BAT, WAT can be remodeled to acquire BAT–like adipocytes now termed as beige adipocytes that display biological features of brown adipocytes such as abundant mitochondria, inducible UCP1 expression and multilocular lipid droplet morphology, under certain circumstances such as cold exposure (Cannon and Nedergaard 2011; Kajimura et al. 2015). Although the beige adipocytes are capable of producing nonshivering thermogenesis through both UCP1–dependent and UCP1–independent mechanisms (Kazak et al. 2015; Bertholet et al. 2017; Ikeda et al. 2017, 2018), the contribution of beige adipocyte thermogenesis to the systemic nonshivering thermogenesis that has been traditionally considered as the major defensive mechanism for the maintenance of body temperature against cold environment is not clear.

The sympathetic nervous system (SNS) directly innervates peripheral fat depots including both BAT and WAT and plays a key role in BAT thermogenesis and WAT lipolysis (Bartness and Song 2007; Barbatelli et al. 2010; Bartness et al. 2010; Vitali et al. 2012; Nguyen et al. 2014). To investigate the physiological significance of beige adipocyte thermogenesis, we limited BAT thermogenesis of the Siberian hamster by denervating SNS to its interscapular BAT (iBAT) and measured core and iWAT and iBAT temperatures (Nguyen et al. 2017). We found that the hamsters with iBAT denervation were surprisingly able to survive and maintain core temperature during acute cold exposure (Nguyen et al. 2017). These animals with defects in iBAT thermogenesis exhibited increased beige adipocyte formation and iWAT temperature (Nguyen et al. 2017), which may compensate for the loss of iBAT heat production. However, it is not clear whether SNS innervation in iWAT is necessary for the compensatory up‐regulation of beige cell formation in animals with impaired BAT thermogenesis.

In the present study, we generated mouse models with a partial chemical denervation of SNS in interscapular BAT (iBAT), inguinal WAT (iWAT), or both, and then assessed beige adipocyte formation with the molecular and immunohistological measurement of UCP1 expression and adipocyte morphology in iWAT.

## Materials and Methods

### Mice

Male C57BL/6J mice were purchased from The Jackson Laboratory (Bar Harbor, ME) for all experiments. Mice were housed in polypropylene cages with a 12‐h light/dark cycle and had free access to water and a normal chow diet. At the end of the studies, mice were euthanized via carbon dioxide inhalation in a euthanasia chamber. All WAT and BAT depots were dissected, weighed, snap‐frozen in liquid nitrogen, and later stored at −80°C or fixed in 10% neutral formalin for further experiments. All animal procedures were approved by the Institutional Animal Care and Use Committee of Georgia State University.

### Chemical denervation of iBAT and iWAT SNS with 6‐hydroxydopamine (6‐OHDA)

SNS denervation was achieved by microinjection of 6‐OHDA (Sigma Aldrich, St. Louis, MO), a selective neurotoxin to SNS nerves (Nguyen et al. 2017), into fat pads that receive SNS innervation. 12‐week‐old male C57BL/6J mice were randomly divided into four experimental groups: (1) Vehicle control in iBAT and Vehicle control iWAT (iBAT‐C & iWAT‐C); (2) 6‐OHDA in iBAT and Vehicle control in iWAT (iBAT‐6‐OHDA & iWAT‐C); (3) Vehicle control in iBAT and 6‐OHDA in iWAT (iBAT‐C & iWAT‐6‐OHDA); (4) 6‐OHDA in iWAT and 6‐OHDA in iBAT (iBAT‐6‐OHDA & iWAT‐6‐OHDA). Mice were anesthetized and iBAT or iWAT depots were exposed by an incision. 6OH‐DA (10 mg/mL) was dissolved in 0.15 mol/L NaCl and 1% ascorbic acid solution and was bilaterally injected into fat depots using a Hamilton syringe at a dose of 100 *μ*g/iBAT lobe or 240 *μ*g/iWAT lobe. Mice were allowed to recover for 7 days and then were subjected to a cold challenge at 5°C for another 7 days.

### Immunohistochemistry and immunoblotting

Adipose tissues were fixed in 10% neutral formalin, embedded in paraffin, cut into 5 *μ*m sections, and stained with UCP1 antibody (1:150, abcam, ab10983) to detect UCP1 protein expression and beige adipocyte formation as we described previously described (Zha et al. 2015; Nguyen et al. 2017). Tyrosine hydroxylase (TH) and UCP1 protein expression in IWAT and IBAT was measured by immunoblotting as we described (Zha et al. 2015; Nguyen et al. 2017). Fat tissues were homogenized in a modified radioimmunoprecipitation assay (RIPA) lysis buffer containing 50 mmol/L Tris‐HCl, 1 mmol/L EDTA, 1% Nonidet P‐40, 0.25% sodium deoxycholate, 150 mmol/L NaCl, 1 mmol/L phenylmethylsulfonyl fluoride, 200 mmol/L Na3VO3, 1% protease inhibitor mixture (Sigma), and 1% phosphatase inhibitor mixture (Sigma). Tissue lysates were separated using SDS‐PAGE. Proteins on the gels were transferred to nitrocellulose membrane (Bio‐Rad, Hercules, CA). The transferred membranes were blocked, washed, and incubated with various primary antibodies, followed by Alexa Fluor 680‐conjugated secondary antibodies (Life Science Techenologies). The blots were developed with a Li‐COR Imager System (Li‐COR Biosciences, Lincoln, NE). The following primary antibodies were used: UCP1 (1:500, abcam, ab23841), TH (1:1000, Millipore, AB152), and *α*‐Tubulin (1:1000, Advanced BioChemicals, ABCENT4777).

### Statistical analysis

All data are expressed as mean ± SE. Differences between groups were analyzed for statistical significance using *t* test or one‐way ANOVA as appropriate. Statistical significance is considered at *P* < 0.05.

## Results

We employed a chemical approach to denervate the iBAT or iWAT SNS by microinjection of 6OH‐DA, a specific neurotoxin to SNS, into the fat pads, because surgical denervation may sever the nerve bundles that contain both SNS and sensory nerves. Using the chemical denervation, we can preserve the sensory nerves that have been shown to interact with SNS (Nguyen et al. 2018) and therefore rule out a potential effect imposed by sensory nerve deficiency. Here we performed a partial chemical denervation of SNS to iBAT, iWAT or both with a vehicle injection as a control. The four groups of mice were established as follows: Control with vehicle injection into both iBAT and iWAT (iBAT‐C & iWAT‐C); iBAT SNS denervation (6‐OHDA) with iWAT intact (iBAT‐6‐OHDA & iWAT‐C); iWAT SNS denervation (6‐OHDA) with iBAT intact (iBAT‐C & iWAT‐6‐OHDA); both iBAT and iWAT denervation (iBAT‐6‐OHDA & iWAT‐6‐OHDA).

**Figure 1:**
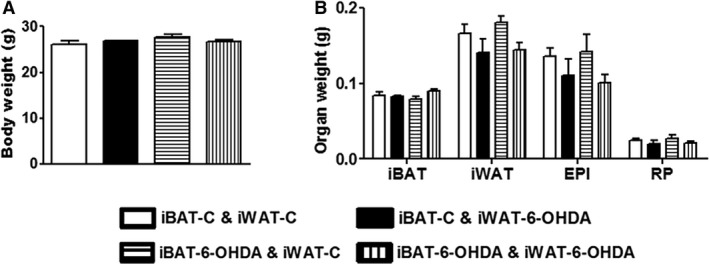
A partial chemical denervation of SNS to iBAT, iWAT or both does not change body weight and fat mass in mice. (A) Body weight in mice receiving a partial chemical denervation of SNS to iBAT, iWAT or both. (B) Fat pad weight in mice receiving a partial chemical denervation of SNS to iBAT, iWAT or both. All data are expressed as mean ± SEM, *n* = 4–8. EPI: Epididymal; RP: Retroperitoneal.

**Figure 2:**
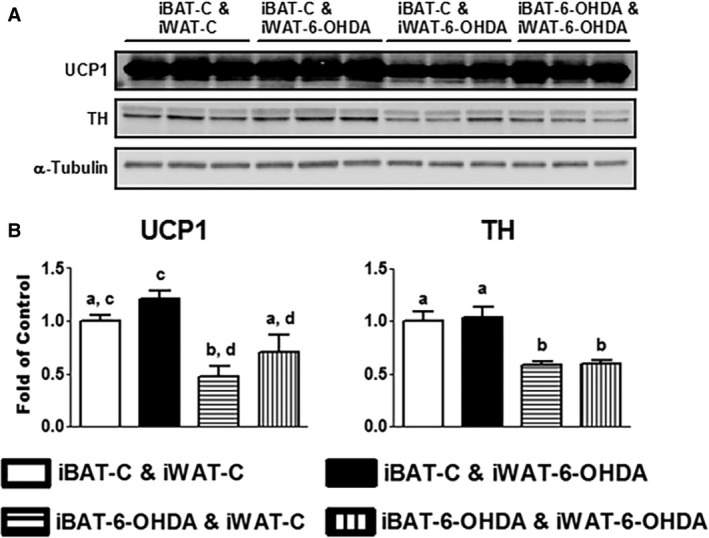
A partial chemical denervation of SNS to iBAT reduces TH and UCP1 protein levels in iBAT. (A) Immunoblots of TH or UCP1 protein. (B) Quantitation of the TH or UCP1 immunoblots. All data are expressed as mean ± SEM, *n* = 4–8. Groups labeled with different letters are statistically different from each other.

**Figure 3:**
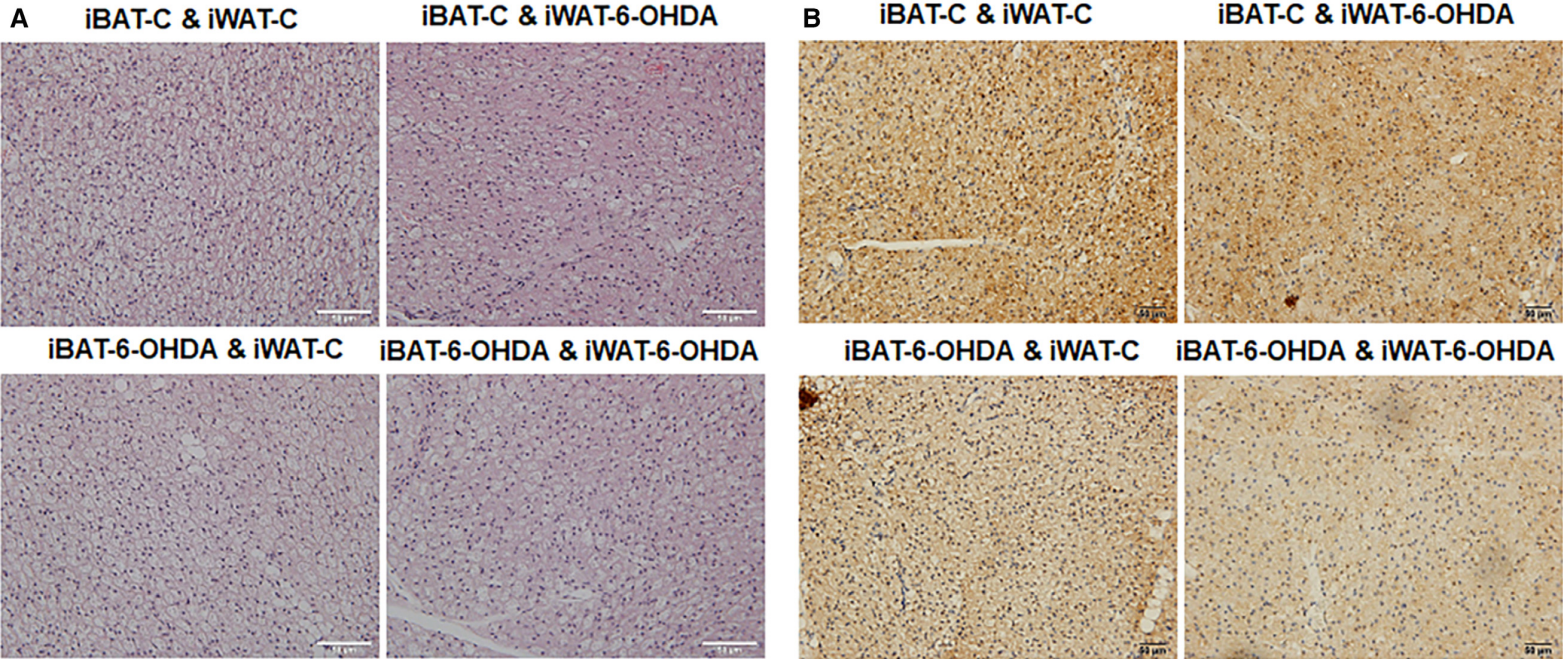
A partial chemical denervation of SNS to iBAT increases BAT cell size and reduces UCP1 protein levels in iBAT. (A) H&E staining of iBAT. (B) UCP1 IHC staining of iBAT.

**Figure 4:**
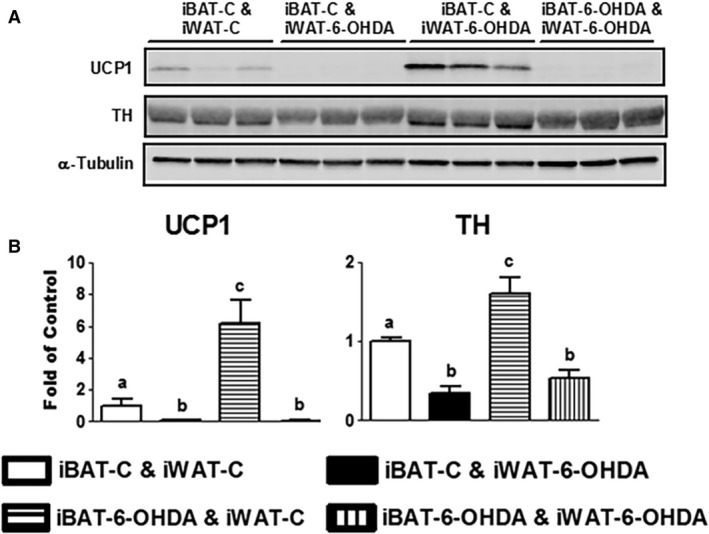
A partial chemical denervation of SNS to iBAT increases TH and UCP1 protein levels in iWAT. (A) Immunoblots of TH or UCP1 protein. (B) Quantitation of the TH or UCP1 immunoblots. All data are expressed as mean ± SEM, *n* = 4–8. Groups labeled with different letters are statistically different from each other.

**Figure 5:**
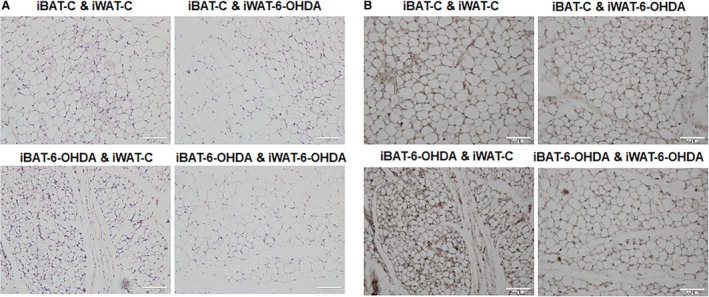
A partial chemical denervation of SNS to iWAT prevents beige adipocyte appearance in iWAT. (A) H&E staining of iWAT. (B) UCP1 IHC staining of iWAT.

All mice receiving denervation surgery survived the 7‐day cold challenge. There was no difference in body weight among all groups of animals after cold exposure, nor was there any difference in fat pad weight (Fig. 1). iBAT SNS denervation alone by microinjection of 6‐OHDA reduced iBAT tyrosine hydroxylase (TH) protein levels by ~50%, indicating a partial SNS denervation in iBAT (Fig. 2). This resulted in more than 50% reduction of UCP1 protein content in iBAT (Fig. 2). In consistence, the partial denervation of iBAT SNS slightly increased brown adipocyte size shown in H&E staining (Fig. 3A) and reduced UCP1 protein in iBAT demonstrated by less UCP1 immuno‐staining (Fig. 3B). Interestingly iBAT SNS denervation increased SNS outflow to iWAT demonstrated by increased iWAT TH protein (Fig. 4), which was associated with marked up‐regulation of UCP1 protein expression in iWAT (Fig. 4). Indeed, SNS denervation in iBAT induced more morphologically distinguished multiocular cells in iWAT shown in H&E staining (Fig. 5A) and UCP1–positive cells demonstrated by immunohistochemical staining (Fig. 5B), indicating an induction of beige cell appearance in iWAT. In contrast, iWAT denervation completely blocked the induction of UCP1 protein expression and beige cell formation in iWAT of mice with iBAT denervation (Figs. 4 and 5).

## Discussion

This study is a logic extension of our prior observations derived from the study of hamsters with iBAT SNS denervation. The animals with impaired brown fat thermogenesis due to SNS denervation were surprisingly normal during a cold challenge (Nguyen et al. 2018). They not only survived the cold challenge but also maintained a normal body temperature (Nguyen et al. 2018). Despite the loss of BAT thermogenesis demonstrated by lower iBAT temperature, these animals were compensated by increased WAT thermogenesis that likely resulted from newly acquired beige adipocytes due to enhanced SNS outflow to WAT (Nguyen et al. 2018). Our present study further confirmed the SNS cross talk between iBAT and iWAT for a functional coordination of SNS outflow, in which SNS innervation to iWAT is necessary for the compensatory induction of beige cells in iWAT due to the defect of BAT thermogenesis caused by SNS denervation. The study demonstrates the necessity of SNS innervation in WAT for beige cell formation.

It has been well recognized that the sympathetic nervous system (SNS) plays a key role in adipose tissue lipolysis and brown fat thermogenesis (Bartness and Song 2007; Barbatelli et al. 2010; Bartness et al. 2010; Frontini and Cinti 2010; Vitali et al. 2012; Nguyen et al. 2014; Ryu et al. 2015). Cold exposure induces the release of catecholamines from the sympathetic nerve terminals in adipose tissue, which then initiates lipolysis in WAT and activates nonshivering thermogenesis in BAT via *β*3‐adrenergic receptor (Ghorbani and Himms‐Hagen 1997; Ghorbani et al. 1997; Klingenspor 2003; Cannon and Nedergaard 2004; Cinti 2005). The brain network that regulates adaptive thermogenesis has been a focus of extensive investigations over years (Bamshad et al. 1998; Shi and Bartness 2001; Cano et al. 2003; Song et al. 2005, 2008; Ryu and Bartness 2014; Ryu et al. 2015) and diverse neural–endocrine pathways have been identified to be involved in the regulation of SNS activation, including the leptin/melanocortin system, the hypothalamic‐pituitary‐thyroid and hypothalamic‐pituitary‐adrenal axis, just to name a few (Rezai‐Zadeh and Munzberg 2013; Munzberg et al. 2016). Despite extensive studies on the neural–endocrine regulation of SNS, much is unknown about whether and how SNS outflow to BAT and WAT is coordinately regulated. Using retrograde pseudorabies viral mapping approach, we recently identified both convergent and divergent central SNS outflow to WAT and BAT, providing neuroanatomical evidence of a cross talk between WAT and BAT SNS (Nguyen et al. 2018). Indeed, when BAT thermogenesis is defective, SNS outflow to WAT is reciprocally activated and drives the formation of beige adipocytes that functionally enhance heat production in WAT compensating for the loss of BAT thermogenesis, thereby maintaining the overall core temperature (Nguyen et al. 2018). It is not clear however what neuronal circuitries relay the signal of BAT thermogenesis deficiency to the enhanced SNS outflow to WAT, which ultimately serves as an impetus for beigeing and thermogenic function in WAT. The cross talk between BAT and WAT for the functional coordination of SNS outflow could be mediated by sensory nerves from the SNS–denervated BAT, which might sense the lower temperature or secretory factors from the defective BAT and project to the brain sites of SNS outflow to WAT. In fact, we recently reported that sensory denervation of WAT alters SNS outflow to BAT (Nguyen et al. 2018), suggesting a role of fat tissue sensory nerve in mediating the coordination of SNS outflow between BAT and WAT. Further studies are warranted to determine the role of BAT sensory nerves in mediating the SNS cross talk between BAT and WAT.

Considering the potential role of BAT sensory nerves in regulation of SNS outflow, we employed the chemical denervation of BAT SNS in order to maintain the integrity of BAT sensory nerves. The surgical denervation that severs the whole nerve bundle containing both sympathetic and sensory nerves cannot distinguish the two and would inevitably damage the sensory nerves. The chemical denervation approach however has its own drawbacks, including incomplete denervation, because animals may not be able to tolerate high dose of 6‐OHDA injection. Indeed, our chemical denervation achieved about 50% of SNS denervation based on the TH protein measurement. The remaining iBAT SNS may complicate the phenotypes. Nonetheless, 50% of SNS denervation in iBAT is sufficient to reduce more than 50% of UCP1 protein expression in iBAT, suggesting an impaired iBAT thermogenic function. Interestingly, this is associated with a marked induction of beigeing in WAT. Although the contribution of beige adipocytes to nonshivering thermogenesis is under debate, mitochondria derived from WAT of mice challenged with cold have comparable UCP1 expression to the mitochondria in traditional BAT and are functional thermogenic (Shabalina et al. 2013). We also found that boosting beigeing indeed raises temperature in iWAT (Nguyen et al. 2018), suggesting a physiological relevance and functional importance of beige adipocyte thermogenesis. This is particularly important, given the fact that functional brown adipocytes in human possess a molecular signature that mirrors beige adipocytes in mice (Sharp et al. 2012; Shinoda et al. 2015). It is conceivable that increasing beige cell thermogenic function through activation of WAT SNS may be a therapeutic strategy to treat obesity (Cypess et al. 2009; van Marken Lichtenbelt et al. 2009; Virtanen et al. 2009).

In sum, we show that the chemical denervation of iBAT SNS down‐regulated UCP‐1 protein expression in iBAT, which was accompanied by an up‐regulation of UCP1 protein expression and enhanced formation of beige cells in iWAT of mice. In contrast, the chemical denervation of iWAT SNS completely abolished the upregulated UCP‐1 protein and beige cell formation in iWAT of mice with iBAT SNS denervation, indicating the physiological significance of WAT SNS for beige cell formation during cold–induced thermogenesis. We conclude that there exists a coordinated thermoregulation for BAT and WAT thermogenesis via a functional cross talk between BAT and WAT SNS.

## Conflict of Interest

None declared.
