# Corrigendum

**DOI:** 10.14814/phy2.14204

**Published:** 2019-08-08

Sympathetic nerve innervation is required for beigeing in white fat

Qiang Cao, Jia Jing, Xin Cui, Hang Shi, Bingzhong Xue

*Physiol Rep, 7 (6), 2019, e14031.*

https://doi.org/10.14814/phy2.14031

In Xue et al. (2019), Figures 2A and 4A has labeling errors.

In Figure 2A, group 3 that was originally labeled as “iBAT‐C & iWAT‐6‐OHDA,” should be corrected as “iBAT‐6‐OHDA & iWAT‐C”.

In Figure 4A, group 3 that was originally labeled as “iBAT‐C & iWAT‐6‐OHDA,” should be corrected as “iBAT‐6‐OHDA & iWAT‐C”.

The authors apologize for the errors.
